# Taking action against medical accidents: A brief history of AvMA and clinical risk management in the NHS

**DOI:** 10.1177/25160435221135120

**Published:** 2022-10-30

**Authors:** Christopher Sirrs

**Affiliations:** Department of History, 2707 University of Warwick, Faculty of Arts Building, University Road, Coventry, CV4 7EQ, UK

**Keywords:** AvMA, clinical negligence, patient safety, medical accidents, NHS, clinical risk management

## Abstract

Established in 1982, Action against Medical Accidents (AvMA)—originally named Action for Victims of Medical Accidents—was effectively the first charity in Britain dedicated to ‘patient safety’. This article provides a historical analysis of the origins and work of AvMA, situating its background in the medical negligence ‘crisis’ of the 1970s and 1980s, growing consumerism in healthcare, and the significant barriers to justice patients confronted following a clinical incident. It also explores AvMA's impacts on evolving attitudes towards patient harm and safety in the NHS. The article asserts that in addition to supporting patients and campaigning for changes in legal procedures, AvMA played an instrumental role in raising the political profile of adverse health events (‘medical accidents’). By supporting claimant solicitors and increasing their chances of legal success, AvMA contributed to the rising tide of negligence claims, which incentivised NHS trusts and health authorities to introduce clinical risk management (CRM). By 2000, CRM was being framed as part of a broader mission to improve quality and safety in healthcare, and AvMA was recognised as a key stakeholder in the new patient safety agenda.

## Introduction

**Figure 1.**
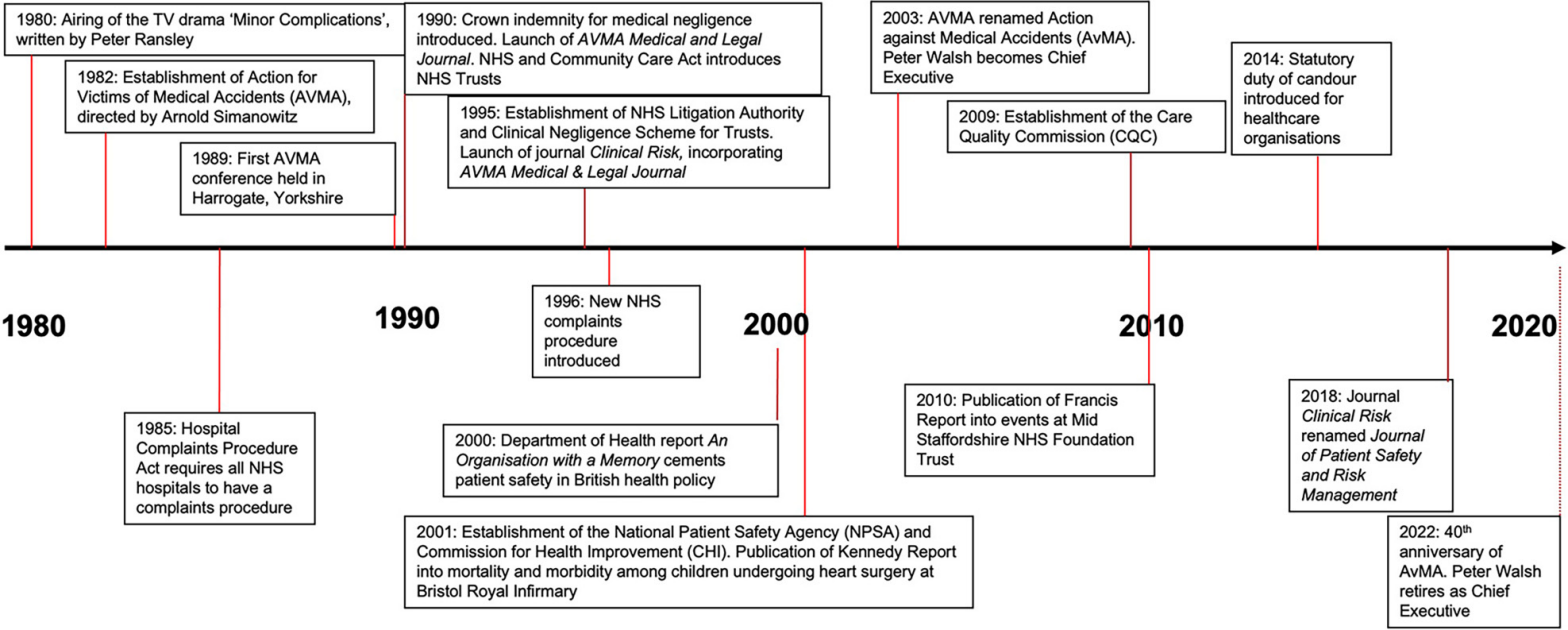
Timeline of historical developments in relation to AvMA, clinical risk management and patient safety in the NHS.

Established in 1982, Action against Medical Accidents (AvMA)—originally named Action for Victims of Medical Accidents—was effectively the first charity in Britain dedicated to ‘patient safety’. This article examines the origins and work of AvMA, situating its background in the medical negligence ‘crisis’ of the 1970s and 1980s, growing consumerism in healthcare, and the significant barriers to justice patients confronted following a clinical incident. It also explores AvMA's impacts on evolving attitudes towards patient harm and safety in the NHS. I argue that in addition to supporting patients and campaigning for changes in legal procedures, AvMA played an instrumental role in raising the political profile of adverse health events (‘medical accidents’). By supporting claimant solicitors and increasing their chances of legal success, AvMA contributed to the rising tide of negligence claims, which incentivised NHS trusts and health authorities to introduce clinical risk management (CRM). By 2000, CRM was being framed as part of a broader mission to improve quality and safety in healthcare, and AvMA was recognised as a key stakeholder in the new patient safety agenda (Figure 1).

## The historical background of AvMA

The claim, commonly expressed by journalists, that clinical negligence claims represent a major threat to healthcare is not new.^1^ Over the twentieth century, figures including doctors, lawyers, and politicians complained that patients were becoming more willing to sue their doctors. The increasing propensity of patients to litigate was noted by bodies including the Medical Defence Union (MDU), which argued in 1988 that ‘[w]henever a doctor or dentist failed to achieve a perfect result, today's patient is likely to consider recourse to law’.^2^

These concerns were not uniquely British. British doctors looked at the level of malpractice claims in the USA with particular unease, fearing the import of an American-style culture of litigation, and alleged defensive medical practices.^3,4^ On the one hand, commentators argued that British society was becoming more litigious in general, and less deferential towards traditional sources of authority such as doctors.^5^ On the other hand, in part due to the emergence of new groups representing patients, such as Community Health Councils (CHCs), it was suggested that patients were becoming better informed and more likely to question their treatment.^6^

There were many complex reasons for the increase in medical negligence claims from the 1970s. Notably, there was the emergence of a more consumerist society in Britain. This encompassed healthcare, where patient groups demanded that closer attention be paid to patients’ rights, choices, and opinions in the planning and running of health services.^7^ There was the advance of medical technology, which resulted in improved outcomes for patients, but also greater risks. As the renowned paediatrician Professor Sir Cyril Chantler remarked in 1999: ‘Medicine used to be simple, ineffective, and relatively safe. Now it is complex, effective, and potentially dangerous’.^8^ At the same time, there was a growing intellectual critique of the power and influence of the medical profession. Commentators ranging from philosophers to lawyers criticised the closed-off and club-like culture of doctors and called upon patients to take greater charge of their own health. The developing field of medical ethics demanded that doctors become more open with patients and cognisant of their intrinsic fallibility.^9–12^ This required, amongst other things, that doctors disclose errors to patients.^13^ The rhetoric of the medical negligence ‘crisis’, however, must be seen alongside the significant cultural, legal, and financial hurdles patients faced when attempting to secure a resolution following a medical injury: whether this entailed compensation, an explanation of what had happened, a promise to prevent recurrence, or even a straightforward apology.

Since the NHS was established in 1948, there had been an ingrained culture of silence and defensiveness around avoidable patient harm. Much of this stemmed from the psychological and emotional impact of mistakes on doctors’ professional identities, their fear of being sued, and its consequences for their reputations.^14,15^ It also stemmed from the fiercely defended culture of clinical autonomy, which meant that doctors’ decisions could not be scrutinised by outside groups, including patients and hospital managers. For instance, whilst medical defence organisations (MDOs) could advise doctors to apologise to patients following a mistake, doctors and hospitals could fail to do so, lest this be seen as an admission of liability.^16,17^ Unlike nurses, doctors rarely participated in the reporting of accidents or ‘untoward occurrences’, fearing that accident reports could come into the hands of solicitors. Clinical error was rarely discussed beyond the professional collegium, and received virtually no academic study.^18^ Even by 2000, virtually nothing was known about the prevalence of adverse events in the NHS.^19–22^

This culture of silence extended to the limited ways in which patients could hold doctors and the NHS to account following an incident. The professional regulator, the General Medical Council (GMC), advised its members as late as the 1970s that errors in diagnosis and treatment were not their concern, and instead focused on other issues, such as alcoholism, sexual misconduct or inappropriate advertising.^23–25^ Formal complaints systems in the NHS were slow to develop, in part due to the concerted opposition of doctors who believed their judgement could be questioned or invite litigation.^7^ The Health Service Commissioner (Ombudsman), which acted as a source of appeal for NHS complainants from 1973, was barred by statute from examining most complaints relating to ‘the exercise of clinical judgement’ until 1996, effectively excluding complaints which related to clinical errors. Even when a coordinated hospital complaints procedure arose from the mid 1980s, the process could prove deeply confusing and frustrating for patients, who were often denied the resolution they needed. Whilst sources of external advice and representation for patients improved, bodies such as Community Health Councils had little legal expertise to help patients with issues relating to medical negligence. Most referred such complex cases directly to solicitors, or after it was established, to AvMA.^26^

The culture of silence around patient harm, and the failure of existing grievance mechanisms, meant that litigation—paradoxically—was often the only avenue for patients and families to hold the NHS accountable following an incident. Yet, litigation itself was highly risky and problematic. The cost of entry for litigation was extremely high. Whilst legal aid was available for some patients, eligibility depended on the receipt of a medical report which determined whether a claim was likely to succeed. However, the cost of obtaining a report was itself unaffordable for many claimants, often amounting to several hundred pounds for an initial report. Many doctors were also reluctant to provide them, since they potentially cast doubt on the competence of their peers.^26^ Even more damagingly, if cases failed, the plaintiff could be expected to pay the defence's costs. This could prove ruinous, with some plaintiffs even forced to sell their homes.

Understandably, due to these financial problems, many patients decided to not pursue litigation at all, or settled early. There was significant dissonance between the media rhetoric around litigation, and the actual chances of a plaintiff's case succeeding at trial: about 1 per cent of cases as of the mid 1970s.^5^

Even if they commenced litigation, patients and families could find it draining and arduous. It was not uncommon for cases to drag on for many years, even over a decade. A common strategy for the defence was to obfuscate for as long as possible in the hope that plaintiffs would run out of money and abandon their cases.^26^ Medical records could go missing, or hospitals would refuse to hand them over. The reports of medical experts could also be routinely withheld. In some cases, such documents would prove there had been no negligence at all—an exercise in futility which only prolonged the legal process.^26^ Critics of the tort system referred to it, justifiably, as a ‘cruel lottery’.^27^

## The formation of AvMA

This is the context which AvMA was established in 1982 as the first charity dedicated to supporting the victims of medical accidents. What is striking about AvMA is that it was prompted by a TV drama.

In November 1980, the BBC aired *Minor Complications*. Written by the playwright Peter Ransley, the drama told the story of Kay Gilbert, an illustrator and divorced mother, who is severely injured when her bowel is punctured during a routine sterilisation. Left disabled and unable to work, the drama followed Kay's battle for compensation, and the enormous challenges claimants in Britain faced when pursuing justice. For instance, without the expertise of a doctor willing to give evidence in her favour, Kay is forced to research the medical literature herself. Denied legal aid, she is forced to pay for her own justice, facing financial ruin (in one scene, Kay struggles to fix a leaking roof due to her financial difficulties). Eventually, with the help of a sympathetic employer, Kay is able to pursue her claim. However, in the dramatic conclusion, Kay discovers that the health authority has chosen not to defend the case. The case does not go to court after all, and she receives an out-of-court settlement. Whilst Kay receives at least some compensation, the health authority continues to deny liability and, ultimately, nothing is learnt from the error.

As the programme began, viewers were informed that the story was based on true events. Ransley based his story on the case of Stella Burnett, a university lecturer injured at Whittington Hospital in London in 1975. Awarded £35,000 out of court, Stella's case had been brought to Ransley's attention by his wife, Cynthia, a social worker at the hospital following an article in the *New Statesman.*^26,28^

The public's overwhelming reaction to *Minor Complications* caught Ransley by surprise. At a time when ‘action lines’ for provocative TV programmes were uncommon, he had inadvertently left his home telephone number with the production desk. Subsequently, as he recalled, ‘the phone started ringing and it never stopped’.^29^ The Ransleys were inundated with phone calls from distressed members of the public who believed that they, or one of their relatives, had suffered a medical injury. Moved by their stories, Ransley placed an advert in *The Guardian* asking for help to establish an organisation dedicated to supporting medical accident victims. A range of sympathetic people responded, including lawyers, a GP, and members of CHCs. In April 1981, they met around the Ransleys’ kitchen table to decide how to proceed. At a press conference in London in July 1982, AvMA was officially launched. Stella Burnett became one of the charity's first trustees.

## The work of AvMA

Despite being founded by a writer, AvMA was first directed by the South African-born lawyer, Arnold Simanowitz, who responded to Ransley's call in 1981 with a desire to get further involved in social issues.^26,30^ The aim of the charity, as declared by its inaugural press release, was ‘to increase awareness of the problem [of medical accidents] both among the general public and within the medical profession’. Amongst its goals was increasing the availability of information for medical accident victims, giving them a voice, and lobbying for improvements in healthcare.

Helping patients to secure compensation, whilst an important goal, was therefore not AvMA's primary aim. AvMA saw litigation as an instrument to facilitate improvements in healthcare, incentivising doctors and managers to make healthcare safer. ‘Action for victims’ for AvMA included not only helping patients secure compensation when warranted, but also supporting them in a holistic sense: helping them to understand their condition, and to receive closure, whether in the form of an explanation, an apology, or remedial treatment. AvMA's work was therefore fundamentally pastoral, reducing distress among patients aggravated by lengthy legal battles. It was also fundamentally cultural, helping to increase the profile of medical accidents in the NHS long before the terms ‘safety culture’ and ‘patient safety’ were in widespread currency.

The close association with AvMA with litigation and compensation was nevertheless a continual source of tension within the charity.^26^ In its inaugural press release and other publications, AvMA emphasised that it only sought to help patients secure the compensation they were entitled to. The word ‘victim’ in the charity's name was debated by trustees, in part because it put the charity on a collision course with doctors, who were ostensibly creating the victims. AvMA also struggled to obtain start-up funding, possibly due the perception it was a group of ‘doctor bashers’. AvMA initially obtained funding from the Greater London Council (GLC), which under the leadership of Ken Livingstone in the 1980s, gained a reputation for political radicalism, supporting numerous community groups.

Yet, AvMA's everyday work with solicitors and its reliance on the legal profession for its operational funding (at times, up to 70 per cent of its income), meant that the charity inevitably became associated with the issues of litigation and compensation.^26^ AvMA established a Lawyers’ Support Group that assisted claimants’ solicitors with handling cases. It organised training courses, conferences, and seminars that improved solicitors’ expertise. The levying of a membership fee for the service—initially twenty-five pounds a year, and later at a higher rate—gave AvMA financial security whilst indicating the support of the legal profession for its goals. Together with a Solicitors’ Referral Service that acted as an approved list of competent legal firms, AvMA heled to create what Simanowitz referred to as a ‘virtuous circle’ by which claimant solicitors gradually accrued experience, success, and ability to take on the well-established defence firms.

At the centre of AvMA's work was its casework with members of the public.^26^ AvMA had a small team of paid caseworkers, some of whom were clinically qualified, such as nurses and paramedics. They were joined by volunteers, some of whom had suffered medical accidents themselves. Caseworkers provided clients with practical support, advising them about their situation and the steps they needed to take. They oversaw the progress of cases which AvMA referred to solicitors. Members of the public contacted AvMA by letter or telephone, and were also referred by bodies such as Community Health Councils. Much later, the charity received funding to set up a helpline which was also staffed by a dedicated mix of volunteers. Both helpline and casework were highly demanding jobs, emotionally and practically. Since the legal bar of negligence was so high, one of the hardest jobs was telling patients (some of whom were undoubtedly motivated by money) they did not have a case—for example, if the injury was the result of a recognised complication.^31^ This could understandably provoke anger and frustration in patients, as a well as accusations that AvMA was ‘part of the system’.

Since medical expertise was central to the determination of negligence, AvMA's relationship with doctors was also crucial.^26^ AvMA helped to arrange reports from sympathetic medical experts prepared to give evidence in court. AvMA advised experts about their role, and ensured that the evidence they gave was sufficiently robust The place of doctors at various times on AvMA's board, and in its pool of medical experts, shows that doctors’ attitudes towards avoidable patient harm was never monolithic. Many genuinely desired to help patients and prise open the medical profession's culture of secrecy. By working with AvMA, they risked being rebuked or censured by their peers. Others, however, were possibly motivated by the money to be made from legal appearances, which could prove to be a lucrative ‘side-hustle’.

## The influence of AvMA

By the 1990s, the position of claimants had significantly improved, with more and more legal firms prepared to support them.^32^ In a sign of how the wider culture around patient harm in the NHS was beginning to change, the number of doctors willing to serve as medical experts also increased. In medical journals such as *The Lancet*, Simanowitz implored doctors to help patients and become more accountable.^33^ However, AvMA's chief executive frequently faced hostility and misapprehension, not least since as claimants’ success increased, doctors viewed AvMA to be driving litigation against them. In one article in *Hospital Doctor*, for instance, AvMA was described as ‘malevolently anti-medical profession’.^26^

It was in AvMA's wider campaigning where much of its impact on safety cultures in the NHS was most visible. AvMA lobbied for changes in legislation and legal procedures to improve patients’ access to justice.^26^ AvMA was successful, for example, in campaigning for changes in legal aid to allow for certificates to be granted for medical reports. This removed yet another financial barrier to litigation and allowed cases to be assessed on their fundamental merits. AvMA also coordinated the solicitors in a key case (*Naylor and others v Preston Area Health Authority & Others* [1987] *2 AER 353*), which ruled that medical reports should be swiftly exchanged between the parties. In the 1990s, AvMA resisted calls to abolish legal aid for clinical negligence cases altogether, although subsequent government cuts have virtually ended the practice, in favour of conditional fee (‘no win no fee’) agreements.

AvMA also lobbied for changes in healthcare, not only to reduce the risk of medical accidents occurring, but to ensure that patients were better supported. For instance, AvMA was a vociferous proponent of a new inspectorate for health standards, a demand that particularly resonated as scandals emerged of poor practices in NHS hospitals (notably, the scandal revolving around paediatric heart surgery at Bristol Royal Infirmary).^34,35^ This demand was ultimately granted by the Labour government, in the form of the Commission for Health Improvement from 2001 (and from 2009, the Care Quality Commission (CQC)). AvMA also campaigned for, and was consulted on new guidance by the NHS on being open with patients following an adverse event.^36,37^ To further this aim, AvMA joined forces with the bereaved father Will Powell to campaign for ‘Robbie's Law’, a statutory duty upon all healthcare professionals to be candid with patients and families after something goes wrong. Building on Powell's work, AvMA succeeded in having a statutory duty of candour implemented in the NHS in 2014. However, to this day, this duty applies only to healthcare organisations registered with the CQC, and not with individual health professionals, who retain only a professional duty.

Arguably, AvMA's greatest influence has been increasing the political profile of ‘medical accidents’ as a policy issue—and thus patient safety, although this term was not widely used before the millennium. From 1989, AvMA organised annual conferences that brought together lawyers, doctors, hospital managers, academics, as well as people interested in the broader social, legal, and ethical dimensions of medical accidents. Through this forum, medical accidents were framed as a problem of safety—as a problem that needed to be corrected through preventive measures—rather than only a legal problem. From 1990, AvMA produced its own journal, the *AVMA Medical & Legal Journal*, which printed articles on medical accidents from a medico-legal perspective and reported important cases. The importance of medical accidents and negligence to British health policy had grown to such an extent that by the mid-1990s, a conference was held on clinical risk management at the Royal Society of Medicine, a clinical risk research unit was set up at University College London, and there were moves to develop a new academic journal, *Clinical Risk* (the predecessor title to the *Journal of Patient Safety and Risk Management*).^38^ In discussions with the journal's editor, the obstetrician Roger Clements, he agreed to incorporate AvMA's own journal within it—an arrangement that continues to this day.^26^ This not only opened up AvMA's work to a new medical audience, but also conferred substantial political legitimacy to the charity. Through its work, therefore, AvMA grew well connected and able to influence health policy. Amongst the charity's notable former trustees include Jean Robinson, former chair of the Patients Association and critic and lay member of the GMC,^25,39^ and Charles Vincent, psychologist and leading patient safety researcher. In 1999, Vincent was a member of the Department of Health expert committee which produced the report *An Organisation with a Memory*, cementing patient safety as an object of British health policy (alongside a special issue of the *BMJ*).^21^ This followed an earlier report by the US Institute of Medicine, *To Err is Human: Building a Safer Health System*, but although the latter was cited in *An Organisation with a Memory*, it does not seem to have directly influenced the committee's formation.^40,41^

By 2000, therefore, AvMA was recognised as a small, but influential stakeholder in what by that point was called ‘patient safety’. Recognition of AvMA's influence came in the form of automatic approval of its Solicitors’ Referral Panel for legal aid cases, funding from the Department of Health, as well as an invitation for Simanowitz to serve on the new National Patient Safety Forum and as a non-executive director of the National Patient Safety Agency (NPSA).^26^

In 2003, Simanowitz was succeeded by Peter Walsh, former chief officer of Croydon CHC and director of the Association of Community Health Councils of England and Wales (ACHCEW).^42^ Under Walsh, AvMA adopted its current name, Action against Medical Accidents, underlining its mission as being about patient safety as well as justice in the widest sense for patients affected by avoidable harm. At the centre of AvMA's work remains its daily helpline support and casework with patients, as well as its Lawyers’ Resource Service. AvMA considers other types of support (such as mediation) where appropriate, and ultimately only around 10 per cent of people it supports go on to take legal action. AvMA arguably remains a leading consumer voice in Britain relating to access to justice in the medico-legal sense. Amongst its recent work is its development of the concept of the ‘Harmed Patient Care Pathway’, which it hopes will transform the way that injured patients and their families are supported by the NHS.^43^

## From clinical risk management to patient safety

It is impossible to distinguish the direct impact of AvMA on rates of medical negligence claims against the NHS. The increase in medical negligence cases long preceded the charity's creation, and was intimately bound with the wider cultural changes described above. It would also be overly simplistic to argue that only AvMA was responsible for emerging interest in patient safety: other factors, such as scandals revolving around poor quality hospital care and poorly performing doctors, were also crucial to the elevation of patient safety as a major policy issue. Nevertheless, by creating a pool of competent solicitors, AvMA influenced the increasing success of claims, and thus contributed to increasing pressure on the NHS to manage clinical risks. Certainly, in part due to the increasing availability of information and support for patients from groups such as AvMA, the number of claims against the NHS continued to rise. Over the 1980s, the number of negligence cases rocketed: in the Oxford health authority region alone, for instance, it is estimated that the frequency of claims increased by 500 per cent over the decade, and the cost of meeting successful claims increased by 250 per cent in real terms.^5^ The costs associated with handling and settling claims also continued to rise. By the mid 1990s, the cost of settling outstanding claims was estimated at £75 million per year, and negligence claims were listed as one of the greatest ‘non-activity’ cost pressures on the NHS budget.^44^ Records also continued to tumble in terms of the cost of individual awards, especially for the victims of brain damage, to provide for their life-long care. For example, in 1987, much ink was spilled over the first £1 million sum awarded to a 21-year-old student, Samir Aboul-Hosn, who suffered brain damage during an operation in London.^45^

Anxiety amongst doctors, lawyers, politicians, and medical figures about medical litigation had a profound effect on attitudes towards patient safety. Firstly, medical accidents and clinical errors became much more visible politically. Prompted by concerns about litigation, for example, scientific research began to be conducted on the causes of medical accidents and their impact on patients and clinicians.^15,18,46^ Secondly, doctors lobbied for the introduction of a ‘no-fault’ system of compensation which would automatically compensate patients, rather than needing them to go to court. Two private members’ bills to this effect were introduced in Parliament in the 1990s, but both failed owing to government concerns it would not reduce costs overall. Since so few medical injuries were actually litigated compared to the actual number of patients suffering injury,^22^ no-fault compensation had the potential to vastly increase the number of compensation claims. AvMA, perhaps surprisingly, opposed no-fault compensation because it believed it did little to address the underlying problem, which was doctors’ accountability. By removing the threat of litigation, Simanowitz suggested, one of the few key levers that made doctors truly accountable to patients would be removed.^47^

Another important development in this period was the decision by the government in 1990 to indemnify NHS hospital doctors against negligence claims, rather than having them to subscribe to a medical defence organisation, as had been the case since 1948. This was an important spur for patient safety, since NHS trusts now had extra financial incentive to invest in systems to reduce adverse events. This was amplified in 1995 by the creation of the Clinical Negligence Scheme for Trusts (CNST). This allowed trusts to pool their risks for negligence claims, and to potentially achieve reductions in their premiums, in exchange for being audited against various standards by the NHS Litigation Authority (now NHS Resolution). For example, trusts were encouraged to establish risk management systems and mechanisms to report and analyse clinical incidents. More broadly, the field of clinical risk management (CRM) developed in this period. Clinical risk managers began to be employed in the NHS to identify, analyse, and control clinical risks, and the NHS Executive provided guidance to hospitals on risk management.^48^ Private companies, such as Datix (established in 1986), also began to develop systems for hospital staff to report and analyse incidents more effectively. Whilst the impetus behind CRM was primarily financial, its supporters, including AvMA, welcomed it as a way to reduce the underlying risks that give rise to patient harm—underlining that AvMA was never purely about promoting compensation.^34^ By the 2000s, CRM was being reframed as part of a broader drive to promote quality and safety in healthcare. For example, risk management (encompassing clinical risk) was listed a key component of ‘clinical governance’, and risk management became part of performance monitoring and standard setting in the NHS.^49^ In this way, CRM—and the financial threat of litigation it responded to—provided a foundation for many of the management processes and systems which are now integral to patient safety.

## Conclusion

Litigation against the NHS continues to be seen as a major issue.^50^ Litigation is increasingly seen as a barrier to the ‘open’ and ‘just’ culture many patient safety advocates seek, by encouraging a hostile and defensive approach to avoidable patient harm.^51^ Recently, for example, calls for no-fault compensation have been renewed by the House of Commons Health Committee,^52^ and already in Wales, the NHS Redress scheme operates, which can approve compensation payments for individuals up to £25,000.^53^ Just a few decades ago, however—in the absence of a wider ‘learning culture’ around adverse events—litigation was often the only lever for patients to hold doctors and the wider NHS accountable for avoidable harm. By advocating for patients, campaigning for changes in legal procedures, and improving the expertise of solicitors, AvMA contributed to the rising tide of medical litigation over the 1980s and 1990s that encouraged the NHS to develop more sophisticated risk management systems. Whilst helping patients to secure compensation was only one part of AvMA's wider work to help patients suffering medical injury, it was this very fear of litigation that was one of the main drivers behind what is now called patient safety.

Recurring scandals around unsafe care in the NHS (most recently, those centring around maternity services at Shrewsbury and Telford, East Kent, and Nottingham) highlight that AvMA's services are required now more than ever. The regulatory and monitoring arrangements for patient safety in the NHS have become more complex in recent years, as new entities have emerged, such as the Healthcare Safety Investigations Branch (soon to be the Health Services Safety Investigations Body) and the Patient Safety Commissioner for England. AvMA will have to navigate this increasingly convoluted and fragmented landscape whilst continuing to offer its unique insights, expertise, and support for patients. As the institutional arrangements for patient safety continue to be in state of flux, the need for a strong and consistent patient voice remains ever present. The fortieth anniversary of AvMA demonstrates the charity's continuing capacity to act as this voice.
